# Optimizing Glioma Resection Outcomes: A Systematic Review of Intraoperative Magnetic Resonance Imaging Guidance in Neurosurgery

**DOI:** 10.7759/cureus.64697

**Published:** 2024-07-16

**Authors:** Thowaiba E Ali, Zarin Nudar Rodoshi, Yoalkris E Salcedo, Vaishvik K Patel, Ismail Khan

**Affiliations:** 1 Medicine and Surgery, University of Khartoum, Khartoum, SDN; 2 Healthcare Administration, University of Tennessee at Chattanooga, Chattanooga, USA; 3 Medical Education, Mymensingh Medical College, Dhaka, BGD; 4 Surgery, Universidad Iberoamericana, Dominican Republic, DOM; 5 School of Medicine, St. George’s University, West Indies, GRD; 6 Internal Medicine, Nishtar Medical University, Multan, PAK

**Keywords:** awake craniotomy, tumor resection outcomes, neurosurgery, glioma resection, intraoperative mri

## Abstract

This systematic review evaluates the efficacy of intraoperative magnetic resonance imaging (iMRI) in enhancing glioma resection outcomes within neurosurgical procedures. Given the complexity and variability of gliomas, achieving precise and safe resections is challenging, necessitating the use of advanced imaging techniques like iMRI. This technology provides real-time, high-resolution insights during surgery, allowing for adaptations based on surgical dynamics and brain shifts. Our comprehensive search across multiple databases selected five significant studies that collectively demonstrate the beneficial impact of iMRI. These studies highlight its role in significantly improving the extent of tumor resection and suggest potential enhancements in both immediate and long-term patient outcomes. The findings indicate that iMRI facilitates more aggressive yet safe resections, particularly in high-risk glioma cases. However, the implementation of iMRI in clinical practice requires careful consideration of training, resource allocation, and the potential variability in outcomes due to study design heterogeneity. Future research should focus on randomized controlled trials to better understand the cost-effectiveness and long-term benefits of iMRI, promoting its wider adoption in neurosurgical settings.

## Introduction and background

Gliomas, representing the most common primary brain tumors, pose significant challenges in neurosurgical management due to their often diffuse infiltration into eloquent brain areas [1]. Achieving maximal safe resection of these tumors is crucial, as it is directly correlated with improved survival rates, reduced recurrence, and better overall prognosis [2]. The standard approach in glioma surgery involves using conventional neuronavigation systems that rely on preoperative magnetic resonance imaging (MRI) to guide the surgical process. However, these systems can be limited by their static nature, using images that do not reflect changes occurring during the surgery.

The intricate relationship between tumor tissue and critical neurological functions complicates the surgical approach. Conventional neuronavigation, while beneficial, is fundamentally constrained because it relies on preoperative imaging data that do not account for the intraoperative brain shift-this is the phenomenon where brain tissues move during surgery due to factors like gravity, loss of cerebrospinal fluid, and the surgical manipulation itself [3,4]. This shift can render preoperative imaging less accurate as the surgery progresses, leading to potential suboptimal resection of the tumor.

Intraoperative magnetic resonance imaging (iMRI) has emerged as a transformative technology in the neurosurgical arena, offering real-time, high-resolution imaging that reflects ongoing surgical manipulations and brain shifts [5]. The use of iMRI aims to facilitate a more aggressive yet safe resection of gliomas by allowing surgeons to visualize tumor boundaries and remaining tumor tissue during the procedure. Previous studies have suggested that iMRI can significantly enhance the extent of tumor resection and may potentially impact postoperative outcomes. However, despite its promising benefits, the adoption of iMRI varies widely, and its effectiveness across different glioma grades and locations remains underexplored [6].

The primary objective of this systematic review is to synthesize existing evidence on the role of intraoperative MRI in improving the outcomes of glioma resection. This review aims to evaluate the efficacy of iMRI in increasing the extent of resection and to assess its impact on immediate postoperative outcomes and long-term survival rates. Compiling and critically analyzing data from various clinical trials and observational studies, this review comprehensively assesses iMRI's value in the current neurosurgical practice, highlighting its benefits, limitations, and potential for routine use in glioma surgeries.

## Review

Materials and methods

Search Strategy

Our search strategy was carefully designed to comply with the Preferred Reporting Items for Systematic Reviews and Meta-Analyses (PRISMA) guidelines, aiming to thoroughly evaluate the use of iMRI for optimizing glioma resection outcomes. We conducted extensive searches across multiple databases, including PubMed, Medline, Embase, the Cochrane Library, and Scopus, spanning from the inception of each database until April 2024. This ensured the inclusion of the most current and pertinent studies.

We employed a combination of keywords and Medical Subject Headings (MeSH) such as "intraoperative magnetic resonance imaging," "iMRI," "glioma," "neurosurgery," and "tumor resection," using Boolean operators like 'AND' and 'OR' to refine the searches. Examples of search strings were "intraoperative MRI AND glioma resection," "iMRI AND neurosurgical outcomes," and "glioma surgery AND real-time imaging." To broaden our search, we also reviewed the reference lists of included articles and searched clinical trial registries and conference proceedings for unpublished or ongoing studies related to iMRI in neurosurgery. The development of this search strategy involved a medical information retrieval expert to ensure precision and thoroughness, focusing only on studies published in English and peer-reviewed journals.

Eligibility Criteria

The eligibility criteria for this systematic review were precisely defined to ensure the inclusion of scientifically sound and relevant studies focusing on using iMRI for glioma resection in neurosurgical practices. We included peer-reviewed articles encompassing clinical trials, observational studies, and retrospective analyses, all evaluating iMRI's role in improving surgical outcomes for glioma patients. These studies specifically detailed the use of iMRI, assessing factors such as the extent of tumor resection, patient survival impact, and postoperative neurological outcomes. To ensure a thorough and high-quality analysis, we considered only articles published in English until April 2024, allowing us to include the most rigorous and recent studies. Eligible studies provided quantitative or qualitative data on the outcomes of iMRI-guided surgeries, especially in patients across various grades of glioma.

Conversely, we excluded studies that did not focus specifically on iMRI in glioma resection or assessed its use outside neurosurgical settings. Non-human research, such as animal studies and grey literature, including conference abstracts and unpublished theses, were omitted to ensure clinical relevance and maintain academic rigor. Additionally, studies that were not published in English or lacked detailed methodological information, such as specifics about the iMRI technique or clear patient outcomes, were excluded. This strategic approach helped maintain a focus on high-quality studies providing direct insights into the effectiveness and safety of iMRI in neurosurgical glioma resection.

Data Extraction

Our data extraction process was meticulously designed to ensure the accuracy and thoroughness of the information gathered for our systematic review of iMRI in neurosurgical glioma resections. Initially, we screened articles by their titles and abstracts, which two independent reviewers assessed for relevance, categorizing them as "relevant," "not relevant," or "potentially relevant." This step was crucial for identifying studies closely aligned with our goal of evaluating iMRI's impact on glioma surgery outcomes.

Articles deemed "potentially relevant" underwent a detailed full-text review using a standardized data extraction form in Microsoft Excel (Microsoft, Redmond, WA, USA). This form, independently used by each reviewer, helped maintain consistency and objectivity. It captured essential data such as the lead author’s name, publication year, study design, population size, significant outcomes of iMRI use, and any noted study limitations or biases. In cases of reviewer disagreement, a senior reviewer resolved discrepancies, ensuring a unified approach to data selection. This meticulous process enabled a comprehensive analysis and robust data synthesis, crucial for substantiating our findings on iMRI's efficacy in glioma resection.

Data Analysis and Synthesis

Given the diversity and variability among the included studies, we chose not to perform a meta-analysis for our review on the use of iMRI in glioma resections. Instead, we adopted a qualitative approach to integrate and interpret the findings, which allowed us to delve into the nuances and specific outcomes of iMRI use in neurosurgical settings. This method thoroughly examined the subtle yet significant aspects of iMRI applications, including their influence on surgical accuracy and patient safety.

The data from each study were categorized to identify common themes and variations in surgical outcomes, technological integration, and patient impacts. This thematic synthesis revealed insights into the operational advantages and limitations of iMRI, enhancing our understanding of its efficacy in glioma resection. We then conducted a narrative synthesis to weave these themes into a cohesive overview of iMRI’s current application in neurosurgery. We discussed the broader implications for clinical practice, highlighted research gaps, and suggested future research directions.

This analytical approach highlighted the commonalities and discrepancies across the studies and enabled a critical assessment of the evidence’s robustness and applicability. Consequently, our review offers a comprehensive perspective on integrating iMRI in glioma surgeries. It underscores its potential to enhance surgical precision and improve patient outcomes while identifying areas ripe for further investigation.

Review

Study Selection Process

**Figure 1:**
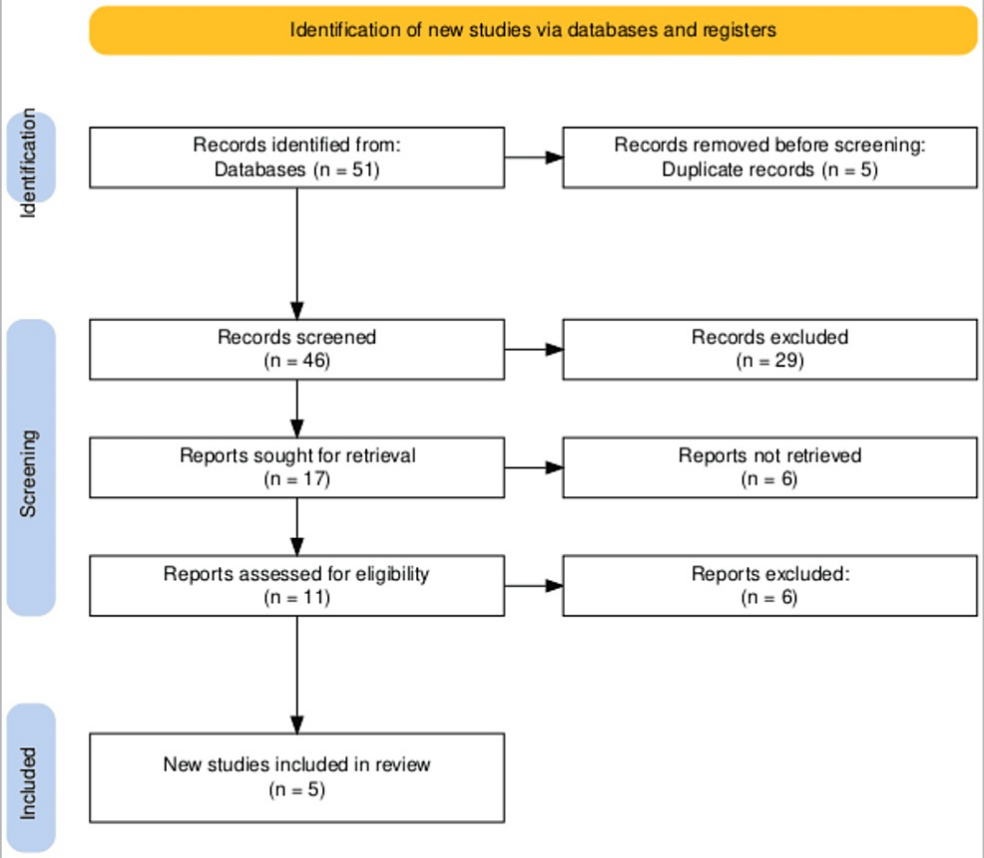
The PRISMA flowchart illustrating studies selection process. PRISMA - Preferred Reporting Items for Systematic Reviews and Meta-Analysis

The search across multiple databases initially identified 51 records. After removing five duplicates, 46 records were screened. This screening process led to the selection of 17 retrieved reports for detailed assessment. Eleven reports were assessed for eligibility, and six were excluded for not meeting the inclusion criteria. Five new studies were ultimately deemed eligible and included in the systematic review. This selection process is succinctly illustrated in the PRISMA flowchart in Figure 1, which methodically outlines the flow from identification through to the inclusion of studies.

Characteristics of the Selected Studies

Our systematic review evaluated five pivotal studies on iMRI in neurosurgical glioma resections. Jackson et al.'s meta-analysis involving 2056 patients indicated that supratotal resections could enhance overall survival compared to gross total resections. Lo et al.'s work showed iMRI's efficacy in increasing gross total resection rates, especially in low-grade gliomas. Tuleasca et al. demonstrated that combining awake craniotomy with iMRI optimizes tumor resection and minimizes neurological deficits. Wach et al.'s analysis highlighted improved resection rates in pediatric surgeries without increasing complications, while Li et al.’s study found iMRI superior to conventional neuronavigation in achieving complete resections and better progression-free survival. These findings underscore iMRI's potential yet also indicate the need for further study due to methodological diversity and variable evidence levels. Details of these studies are provided in Table 1.

**Table 1:** The table summarizing the principle studies included in the article. SpTR: Supratotal Resections GTR: Gross Total Resections OS: Overall Survival GBM: Glioblastoma RCTs: Randomized Controlled Trials OBS: Observational Studies RRs: Risk Ratios CI: Confidence Interval PFS: Progression-Free Survival LOS: Length of Surgery iMRI: Intraoperative Magnetic Resonance Imaging IoMRI: Intraoperative MRI PRISMA: Preferred Reporting Items for Systematic Reviews and Meta-Analyses GTR: Gross Total Resection SSIs: Surgical Site Infections OR: Odds Ratio

Lead Author	Purpose	Methods	Results	Conclusion	Study Quality	Comments
Christina Jackson et al. [7]	To compare the impact of supratotal resections (SpTR) versus gross total resections (GTR) on the overall survival (OS) of glioblastoma (GBM) patients.	Systematic review and meta-analysis on databases including PubMed, Embase, The Cochrane Library, Web of Science, Scopus, and ClinicalTrials.gov focusing on studies comparing OS after SpTR versus GTR.	From 8902 citations, 11 articles met inclusion criteria covering 2056 patients (810 underwent SpTR). 9 of 11 studies showed improved OS with SpTR (median OS improvement of 10.5 months), with no significant difference in postoperative complications. Subgroup analysis showed a 35% lower mortality risk in SpTR versus GTR.	SpTR may offer improved OS over GTR for GBM patients, particularly with anatomical SpTR, but results are limited by study variability and heterogeneity. Prospective clinical trials are needed.	Variable, with ten studies presenting level IV evidence and one study presenting level IIIb evidence.	The findings are promising for SpTR but need confirmation in well-designed prospective studies due to the significant heterogeneity and variable quality of the included studies.
Yu Tung Lo et al. [8]	To evaluate the benefits of intraoperative magnetic resonance imaging (iMRI) in surgeries for high-grade gliomas (HGGs) and low-grade gliomas (LGGs).	Meta-analysis of RCTs and observational studies (OBS) until November 29, 2018, focusing on the use of iMRI versus conventional surgery. Pooled risk ratios (RRs) or hazard ratios were evaluated.	15 articles included (3 RCTs, 12 OBS). RCTs showed higher gross total resection (GTR) with iMRI (RR, 1.42; 95% CI, 1.17-1.73), especially in LGGs. OBS showed improvements in GTR and extent of resection (EOR) with iMRI across all glioma types. No significant impact on progression-free survival (PFS), overall survival (OS), or length of surgery (LOS).	iMRI improved GTR rates in glioma surgery, particularly in LGGs, but did not show benefits in PFS or OS.	Mixed, with three RCTs and twelve observational studies.	The study supports the use of iMRI for improving surgical outcomes in terms of resection rates but underscores the need for more definitive evidence regarding long-term survival benefits.
Constantin Tuleasca et al. [9]	To evaluate the impact of combining awake craniotomy and intraoperative MRI (IoMRI) on surgical outcomes in patients with primary brain tumors.	Systematic review following PRISMA guidelines, including 13 series totaling 527 patients and 541 surgeries. Focus on operative time, intraoperative seizures, rates of initial and final complete resection, and postoperative complications.	The mean surgery duration was 6.3 hours. The intraoperative seizure rate was 3.7%. The initial complete resection rate at first IoMRI was 35.2%, and 46% of patients underwent additional resections after IoMRI. The final GTR rate at discharge was 56.3%. The rate of immediate postoperative complications was 27.4%, and permanent complications were 4.1%.	Combining awake craniotomy with IoMRI can significantly enhance the resection of brain tumors in critical areas, with manageable technical challenges and evident benefits for preserving neurological function.	The study involves multiple series and provides a comprehensive overview.	The article highlights the clinical benefits of advanced surgical techniques in neurosurgery, stressing the importance of experienced teams and suggesting that further randomized trials may not be essential.
Johannes Wach et al. [10]	To analyze the impact of intraoperative MRI (iMRI) on pediatric brain tumor surgery.	Systematic review and meta-analysis using MEDLINE/PubMed with specific search terms related to pediatric brain tumor surgery and iMRI. The review identified 126 potential publications, with 11 meeting the inclusion criteria.	The overall mean rate of GTR in pediatric low-grade and high-grade gliomas was 78.5%, with specific rates for low-grade gliomas at 74.3%. The rate of SSIs was low at 1.6%. Transient postoperative neurologic deficits were observed in 33.0% of patients.	iMRI-guided surgery appears to enhance the extent of resection in pediatric glioma surgery while maintaining standard rates for SSIs and neurologic deficits post-surgery.	Involves a moderate number of studies and patients, indicating a robust meta-analysis.	The findings underscore the utility of iMRI in pediatric brain tumor surgeries, especially in improving surgical outcomes without significantly increasing complication rates.
Ping Li et al. [11]	To evaluate the benefits of intraoperative MRI (iMRI) over conventional neuronavigation-guided resection in patients with gliomas.	Systematic review and meta-analysis of RCTs, two-arm prospective studies, and retrospective studies using databases such as Medline, PubMed, Cochrane, and Google Scholar up to September 26, 2015.	iMRI was associated with a higher rate of gross total resection compared to conventional neuronavigation (OR 3.16; CI 2.07-4.83, P < .001). iMRI was linked to a higher progression-free survival rate (OR 1.84; CI 1.15-2.95; P = .012) but not in overall survival (OS).	iMRI-guided surgeries in glioma patients lead to more complete resections and improved progression-free survival but not overall survival. The study highlights the need for more robust trials.	The study uses a mix of RCTs and non-RCTs, indicating a breadth of data but also a potential for variable quality.	The results support the use of iMRI for potentially enhancing surgical outcomes in glioma resections, though further high-quality studies are needed to confirm long-term survival benefits.

Discussion

Our systematic review reveals that iMRI significantly enhances the extent of tumor resection in glioma surgeries, a finding consistently supported across several studies, including those by Tuleasca et al. [9] and Wach et al. [10]. These studies demonstrate that iMRI facilitates a higher rate of gross total resection (GTR), with Tuleasca et al. reporting improved surgical outcomes in critical brain areas and Wach et al. showing a 78.5% GTR rate in pediatric glioma surgeries. The use of iMRI has been instrumental in allowing surgeons to achieve a more comprehensive removal of tumor tissues while preserving crucial neurological functions, thus contributing to improved immediate postoperative outcomes and potentially reducing the rates of recurrence [12].

Comparatively, these outcomes mark a substantial improvement over baseline standards, where conventional neuronavigation often falls short due to intraoperative brain shift and other dynamic changes. For instance, the study by Li et al. [11] indicates a higher rate of complete resections and improved progression-free survival with iMRI compared to traditional methods, highlighting the technology’s impact on surgical precision and patient management. This shift in practice underscores the direct benefits of integrating advanced imaging techniques into neurosurgical procedures and aligns with broader trends aimed at enhancing the precision of medical interventions in complex oncological cases [13].

The findings from our systematic review align with the broader body of existing literature that supports the efficacy of iMRI in enhancing glioma resection outcomes. Studies such as those conducted by Lo et al. [8] and Li et al. [11] corroborate the advantage of iMRI in achieving superior surgical precision, with Lo et al. [8] highlighting the improved gross total resection rates, particularly in low-grade gliomas. These results resonate with the growing consensus in the neurosurgical community about the utility of iMRI in providing real-time feedback during surgeries, thereby allowing for adjustments that optimize tumor removal while minimizing collateral damage to surrounding healthy tissues. However, there are discrepancies in the literature, particularly concerning the long-term benefits of iMRI, such as overall survival rates [14]. For example, while our review and the studies by Lo et al. and Li et al. noted improvements in immediate surgical outcomes, the translation of these benefits into long-term survival gains remains inconsistently reported, suggesting variability possibly influenced by differences in tumor pathology, patient demographics, or even specific surgical techniques and iMRI equipment used.

The mixed outcomes reported in previous studies may also reflect methodological variations, such as differences in study design (retrospective vs. prospective), the definition of outcome measures, or the inclusion criteria for patient populations. For instance, some studies might include patients with different glioma grades or varying extents of tumor infiltration, which can significantly influence the prognosis and response to surgery, irrespective of imaging technique. Integrating our findings with this varied landscape highlights the importance of considering these factors when interpreting the efficacy of iMRI in clinical practice. This contextual understanding prompts further investigation into standardized protocols and techniques to harness the full potential of iMRI in neurosurgery [15].

The incorporation of iMRI into neurosurgical procedures for glioma resection carries profound implications for clinical practice [16]. By significantly enhancing the extent of tumor resection, as demonstrated in our systematic review, iMRI allows neurosurgeons to operate with a higher level of precision, potentially reducing recurrence rates and improving patient prognoses [17]. This technological advancement necessitates a shift in surgical strategies, emphasizing a more dynamic approach that can adapt to real-time feedback during operations. Consequently, iMRI may influence patient selection criteria, favoring its use in cases involving complex, high-grade gliomas or tumors near critical brain areas where maximal resection with minimal functional disruption is crucial [18].

The successful integration of iMRI technology also demands significant training and resource allocation changes within healthcare settings. Neurosurgeons will require specialized training to effectively interpret iMRI data and make intraoperative decisions based on this information [19]. This training should extend beyond technical knowledge, including decision-making processes that leverage real-time imaging to guide surgical interventions. Furthermore, adopting iMRI technologies implies substantial investment in specialized equipment and modifications to existing surgical infrastructure to accommodate the hardware and workflows associated with iMRI [20]. Hospitals and medical centers will need to evaluate the cost-effectiveness of these investments in terms of improved surgical outcomes and potential reductions in long-term treatment costs associated with recurrent surgeries. As such, while the benefits of iMRI are clear, its implementation poses logistical and financial challenges that must be carefully considered by healthcare providers and administrators [21].

While comprehensive, our systematic review has limitations that could impact the interpretation of the findings. One significant limitation is the heterogeneity in study designs among the included studies, which ranged from observational studies to controlled trials. This diversity can introduce variability in outcomes and potentially bias the conclusions drawn. Additionally, the possibility of selection bias cannot be disregarded, as studies with favorable outcomes might be more likely to be published. To mitigate these issues, we employed a rigorous methodological framework for selecting studies and extracting data, adhering to PRISMA guidelines to ensure transparency and replicability [22]. We also sought to include a broad range of studies to capture a comprehensive view of the field, and where possible, we conducted subgroup analyses to understand the impact of study design on the results [23].

The findings of our review highlight several areas for future research that could further refine the use of iMRI in neurosurgery. There is a clear need for more randomized controlled trials (RCTs) that rigorously evaluate the outcomes of iMRI-assisted glioma surgeries, particularly focusing on long-term patient outcomes such as survival rates and quality of life post-surgery [24]. Future studies should also explore the cost-effectiveness of iMRI to address whether the benefits in surgical outcomes justify the substantial investment in technology and training [25]. Additionally, as iMRI technology advances, new studies could investigate integrating augmented reality and machine learning algorithms to enhance the precision and utility of real-time imaging during complex surgical procedures. Such advancements have the potential to not only improve the accuracy of tumor resection but also reduce the cognitive load on surgeons, leading to better patient outcomes.

## Conclusions

The findings of this systematic review underscore the transformative potential of iMRI in the neurosurgical resection of gliomas. By enhancing the precision and extent of tumor removal, iMRI represents a pivotal advancement in neurosurgical techniques, significantly improving patient outcomes and quality of life. Integrating iMRI into routine clinical practice promises to advance the standard of care for patients with gliomas. It sets a new benchmark for surgical precision that could extend to other areas of neurosurgery. As we move forward, the neurosurgical community must embrace these innovations, supported by ongoing research and training, to fully realize the potential of iMRI in improving prognoses and reducing the burden of brain tumors on patients worldwide.
